# Integration of digital phenotyping, GWAS, and transcriptomic analysis revealed a key gene for bud size in tea plant (*Camellia sinensis*)

**DOI:** 10.1093/hr/uhaf051

**Published:** 2025-02-20

**Authors:** Shuran Zhang, Si Chen, Zhilu Fu, Fang Li, Qiyu Chen, Jianqiang Ma, Yuanquan Chen, Liang Chen, Jiedan Chen

**Affiliations:** Key Laboratory of Biology, Genetics and Breeding of Special Economic Animals and Plants, Ministry of Agriculture and Rural Affairs, Tea Research Institute of the Chinese Academy of Agricultural Sciences, Meiling South Road, Hangzhou 310008, China; Key Laboratory of Biology, Genetics and Breeding of Special Economic Animals and Plants, Ministry of Agriculture and Rural Affairs, Tea Research Institute of the Chinese Academy of Agricultural Sciences, Meiling South Road, Hangzhou 310008, China; Key Laboratory of Biology, Genetics and Breeding of Special Economic Animals and Plants, Ministry of Agriculture and Rural Affairs, Tea Research Institute of the Chinese Academy of Agricultural Sciences, Meiling South Road, Hangzhou 310008, China; Key Laboratory of Biology, Genetics and Breeding of Special Economic Animals and Plants, Ministry of Agriculture and Rural Affairs, Tea Research Institute of the Chinese Academy of Agricultural Sciences, Meiling South Road, Hangzhou 310008, China; Key Laboratory of Biology, Genetics and Breeding of Special Economic Animals and Plants, Ministry of Agriculture and Rural Affairs, Tea Research Institute of the Chinese Academy of Agricultural Sciences, Meiling South Road, Hangzhou 310008, China; Key Laboratory of Biology, Genetics and Breeding of Special Economic Animals and Plants, Ministry of Agriculture and Rural Affairs, Tea Research Institute of the Chinese Academy of Agricultural Sciences, Meiling South Road, Hangzhou 310008, China; Guangxi South Subtropical Agricultural Sciences Research Institute, Longzhou County, Chongzuo 532415, China; Key Laboratory of Biology, Genetics and Breeding of Special Economic Animals and Plants, Ministry of Agriculture and Rural Affairs, Tea Research Institute of the Chinese Academy of Agricultural Sciences, Meiling South Road, Hangzhou 310008, China; Key Laboratory of Biology, Genetics and Breeding of Special Economic Animals and Plants, Ministry of Agriculture and Rural Affairs, Tea Research Institute of the Chinese Academy of Agricultural Sciences, Meiling South Road, Hangzhou 310008, China

## Abstract

Tea plant (*Camellia sinensis*) is among the most significant beverage crops globally. The size of tea buds not only directly affects the yield and quality of fresh leaves, but also plays a key role in determining the suitability of different types of tea. Analyzing the genetic regulation mechanism of tea bud size is crucial for enhancing tea cultivars and boosting tea yield. In this study, a digital phenotyping technology was utilized to collected morphological characteristics of the apical buds of 280 tea accessions of representative germplasm at the ‘two and a bud’ stage. Genetic diversity analysis revealed that the length, width, perimeter, and area of tea buds followed a normal distribution and exhibited considerable variation across natural population of tea plants. Comparative transcriptomic analysis of phenotypic extreme materials revealed a strong negative correlation between the expression levels of four *KNOX* genes and tea bud size. A key candidate gene, *CsKNOX6*, was confirmed by further genome-wide association studies (GWAS). Its function was preliminarily characterized by heterologous transformation of *Arabidopsis thaliana*. Overexpression of *CsKNOX6* reduced the leaf area in transgenic plants, which initially determined that it is a key gene negatively regulating bud size. These findings enhance our understanding of the role of *KNOX* genes in tea plants and provide some references for uncovering the genetic regulatory mechanisms behind tea bud size.

## Introduction

Tea plant (*Camellia sinensis*) is one of the most important beverage crops in the world, cultivated in >60 countries and regions worldwide, with >2 billion people drinking tea globally (International Institute for Sustainable Development, 2024). In high-quality tea production, leaves are typically harvested according to standards such as one bud, one bud with one leaf, and one bud with two leaves [1]. The size of tea buds not only directly affects the yield and quality of fresh leaves but is also closely related to the suitability of the tea for processing [2]. Different types of tea have varying shapes and different requirements for bud and leaf size [3]. For a long time, research on the molecular mechanisms regulating the size of tea buds and leaves has been very limited, severely restricting genetic improvement efforts for this trait. Analyzing the genetic regulatory mechanisms of tea bud size has potential significance for improving tea plant cultivars and increasing tea yields.

The formation of plant bud and leaf morphology is a complex physiological and biochemical process, primarily involving the initiation and development of leaf primordia, establishment of leaf polarity, and ultimately, leaf growth and differentiation [4]. Each of these processes is regulated collectively by transcription factors (TFs), plant hormones, and microRNAs (miRNAs) [5]. The growth of plant leaves to a specific size is primarily determined by developmental signals [6]. The development of leaf primordia begins at the shoot apical meristem (SAM), where two classes of homeodomain (HD) TFs, WUSCHEL (WUS) and Class I KNOTTED1-like homeobox (KNOXI), play crucial roles in the formation and maintenance of meristematic tissue [7]. Current research has revealed two conserved mechanisms for the initiation of leaf primordia: the first involves the antagonistic relationship between *KNOXI* and the *ARP* genes, including *ASYMMETRIC LEAVES1* (*AS1*) / *ROUGH SHEATH2* (*RS2*) / *PHANTASTICA* (*PHAN*), which encode a type of MYB-domain TFs, promoting the differentiation of leaf primordia [8–10], while the second relies on the polar transport of auxin by transport proteins PIN-FORMED1 (PIN1) and AUXIN1 (AUX) / LIKE AUXIN1 (LAX), ensuring that auxin is transported to the initial position of leaf primordia development to induce differentiation [11]. After the development of leaf primordia, most plants begin to establish polarity along the 3D spatial axes, a process primarily regulated by antagonistic relationship among TFs. Leaf adaxial–abaxial polarity is maintained by domain-specific TFs. On the adaxial side of the leaf, the AS1–AS2 complex inhibits the expression of the *KNOXI* gene, along with the functionally redundant *REVOLUTA* (*REV*) / *PHABULOSA* (*PHB*) / *PHAVOLUTA* (*PHV*), which encodes HD-ZIPIII TFs to promote the establishment of adaxial surface [12–14]. The establishment of abaxial side depends on the activity of KANAD (KAN), YABBY (YAB), and AUXIN RESPONSE FACTOR (ARF) TFs. *KAN* and *HD-ZIPIII* antagonize each other, while *AS1*/*AS2* inhibit the expression of *KAN* and *ARF3*/*ARF4* [15]. The width of the leaf is determined by growth along the mediolateral axis. The two most important regulators are encoded by *WUSCHEL-LIKE HOMEOBOX* (*WOX*) and *PRESSED FLOWER* (*PRS*). The expression of *WOX1*/*PRS* in the middle of the leaf primordia is inhibited by *KAN*, while *YAB* promotes *WOX1*/*PRS* expression [16]. The length of the leaf is determined by growth along the proximal–distal axis. The three genes *BLADE*-*ON*-*PETIOLE1* (*BOP1*) / *BOP2*, *ROTUNDIFOLIA3* (*ROT3*) / *ROT4*, and *LONGIFOLIA1* (*LNG1*) / *LNG2* redundantly regulate proximal–distal polarity [17, 18]. Following polarity establishment, the leaf primordium begins to undergo cell proliferation and cell expansion. The precise regulation of these two growth stages determine the final size of the leaf [19].

To date, numerous studies have indicated that KNOX is a class of key TFs that regulate plant growth, development, and morphological establishment [20]. They possess a conserved DNA-binding domain and are widely distributed across various plant species [21]. KNOX belongs to the THREE AMINO ACID EXTENSION (TALE) superfamily and typically forms heterodimers with BELL proteins to perform its functions [22]. The maize *KNOETTED1* (*KN1*) gene is the first *KNOX* gene identified in plants [23]. In Arabidopsis, nine *KNOX* genes have been cloned, which can be classified into three categories based on sequence similarity, intron positions, expression patterns, and phylogenetic analysis [10, 24, 25]. The *KNOXI* includes four genes: *SHOOTMERISTMELESS* (*STM*), *KN1-LIKE* in *Arabidopsis thaliana1* (*KNAT1*) / *BREVIPEDICELLUS* (*BP*), *KNAT2*, and *KNAT6*, which are primarily expressed in meristematic tissues [26]. The *KNOXII* includes four genes: *KNAT3*, *KNAT4*, *KNAT5*, and *KNAT7* [27]. The *KNATM* consists only of the *KNATM* gene [28]. Except for KNATM, which contains only KNOX1 and KNOX2 domains, other KNOX proteins possess four characteristic conserved domains: KNOX1, KNOX2, ELK, and KN HDs [29]. The *KNOXI* gene plays a critical role in the formation and maintenance of the SAM. Among them, *STM* is expressed throughout the SAM and is essential for maintaining normal stem cell development. Mutations in *STM* result in the inability of the apical meristem to produce new shoots, leading to the termination of bud growth [30]. *KNAT1* and *KNAT2* are also expressed in the SAM, but they are mainly expressed in the Peripheral Zone (PZ) [31]. *KNAT6* and *KNAT2* exhibit high homology and play important roles in the development of inflorescences in Arabidopsis [32]. Additionally, leaf shape is closely related to the expression pattern of *KNOXI*; overexpressing of *KNAT1* in Arabidopsis results in deeper leaf serrations on the leaf margins [33]. In contrast, research on *KNOXII* is relatively limited; it is currently believed that *KNOXII* can inhibit the expression of *KNOXI*, thereby promoting leaf growth and development [34].

In this study, we collected images of the apical buds from 280 accessions of representative tea germplasm at the one bud with two leaves (‘two and a bud’) stage. Using digital phenotyping techniques, we identified the bud size phenotypes and conducted a genetic diversity study. The molecular mechanism underlying the regulation of tea bud size was investigated by RNA-seq, revealing that *KNOX* genes might be potential regulators of tea bud size. Further analysis through genome-wide association studies (GWAS) confirmed that *CsKNOX6* may serve as the key candidate gene, and we conducted preliminary functional validation through heterologous transformation in Arabidopsis.

## Results

### Statistical analysis of bud size traits based on image feature in 280 tea accessions

**Figure 1:**
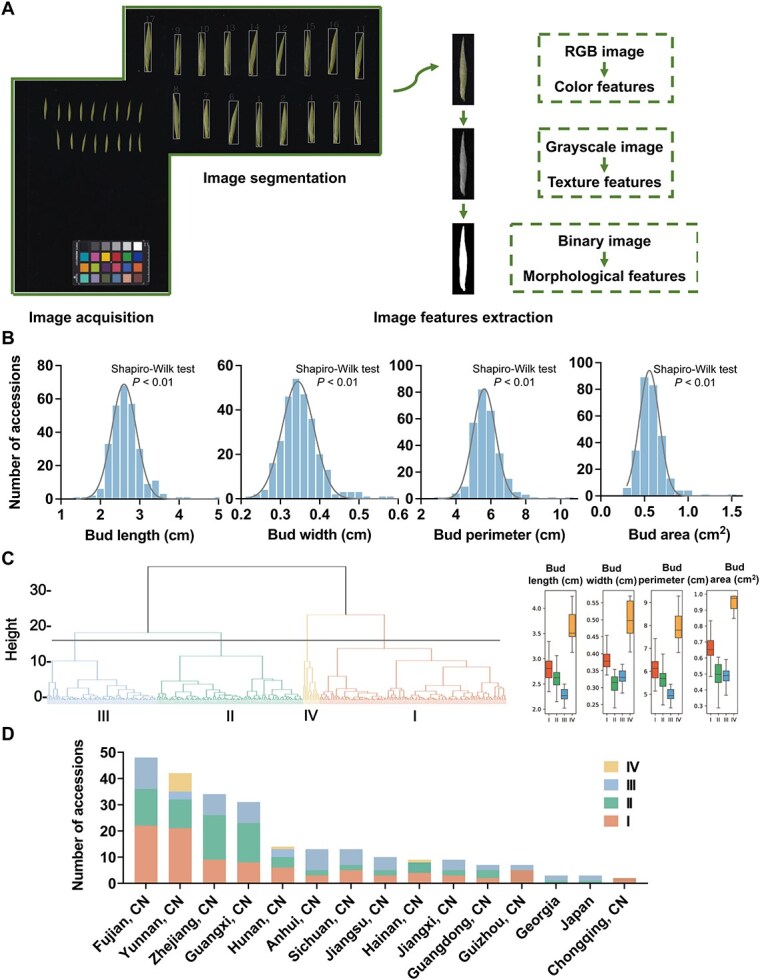
**Statistical analysis of bud traits based on image collection and feature extraction in 280 tea accessions.** (A) Image collection and feature extraction process. (B) Frequency distribution of bud length, width, perimeter, and area. (C) Cluster analysis of 280 tea accessions based on bud size phenotype and the trait distribution for each group. (D) Grouped statistical chart of the top 15 regions by accession numbers ranking.

To quickly and accurately obtain the phenotypic traits of tea buds, this study employed image processing technology (Fig. 1A) and systematically collected images of the tea apical buds. We extracted four image features: bud length, bud width, bud perimeter, and bud area, and conducted a comprehensive identification of bud size phenotypes in 280 tea accessions. As shown in Fig. 1B, each image feature exhibited considerable variation and followed a normal distribution within the natural population (Shapiro–Wilk test; *P* < 0.01). The coefficient of variation (CV) for the four image features ranges from 14.45% to 24.74%, with an average of 17.30%. Among them, the bud area exhibited the greatest variation, with a range of 0.27–1.46 cm^2^. The diversity index (*H*′) ranges from 1.93 to 1.97, with an average of 1.95. The CVs for all image features are >10%, and the diversity indices are all >1.9, indicating that bud size traits exhibit extensive variation in natural populations and possess rich genetic diversity. In addition, the broad-sense heritability of the four phenotypic traits ranges from 0.60 to 0.90, indicating that at least 60% of the variation in bud size phenotypes is determined by genotypic variation. This trait is relatively stable during the genetic process and is less influenced by the environment (Table 1), suggesting that the associated genes regulating bud size in tea plants are promising for use in variety improvement.

**Table 1:** Image features descriptive statistics of bud size among 280 tea accessions

**Image feature**	**Max**	**Min**	**Mean ± SD**	**CV (%)**	***H*′**	***h*** ^**2**^
Bud length	4.91	1.46	2.65 ± 0.38	14.53	1.97	0.72
Bud width	0.71	0.22	0.35 ± 0.05	15.46	1.93	0.90
Bud perimeter	10.62	3.23	5.75 ± 0.83	14.45	1.95	0.71
Bud area	1.48	0.27	0.58 ± 0.14	24.74	1.95	0.60

To further screen for specific germplasm, we conducted a cluster analysis on the natural population. The results indicate that at a Euclidean distance of 16, the 280 tea accessions can be categorized into four groups (Fig. 1C). Group I includes 114 accessions, with moderate bud size. The average bud perimeter is 6.14 cm, and the average bud area is 0.66 cm^2^. This group has the richest source of resources, with the highest number originating from Fujian and Yunnan. Group II includes 89 accessions, with an average bud perimeter of 5.61 cm and an average bud area of 0.49 cm^2^. Over 60% of the materials in this group come from Zhejiang, Guangxi, Fujian, and Yunnan. Group III includes 67 accessions, all of which are small bud resources, with an average bud perimeter of 4.92 cm and an average bud area of 0.48 cm^2^. More than 50% of the materials in this group come from Fujian, Anhui, Zhejiang, and Guangxi. Group IV includes 10 accessions, all of which are large bud resources, with an average bud perimeter of 8.12 cm and an average bud area of 1.02 cm^2^. Among these, seven accessions come from Yunnan, and one accession comes from Hainan, all of which are *C. sinensis* var. *assamica*. The grouped statistical chart of the top 15 regions by accession numbers ranking was shown in Fig. 1D. The specific grouping information was provided in Table S1.

### Transcriptome analysis of extreme accessions for tea bud size

**Figure 2:**
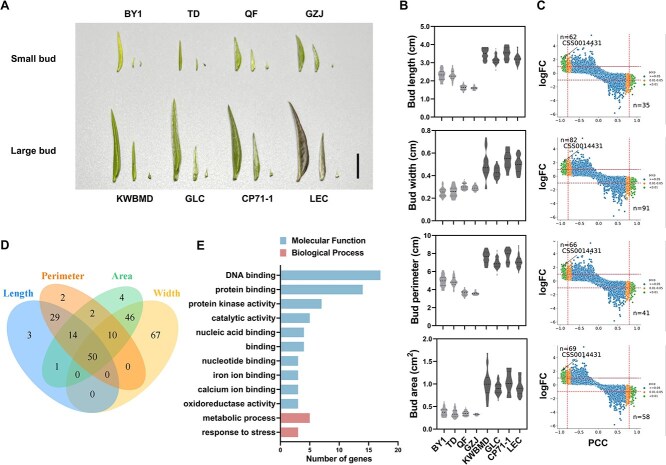
**RNA-seq analysis of bud size in tea plant.** (A) Anatomy diagram of tea bud in eight selected accessions. Black bar = 1 cm. (B) Violin-plot distributions of bud length, width, perimeter, and area in eight selected accessions. (C) Gene screening based on Pearson correlation analysis on four traits. (D) Venn diagram of correlation genes for four traits. (E) GO enrichment analysis of 50 genes associated with bud size.

Transcriptome analysis serves as an effective tool for identifying candidate genes. To identify and analyze key genes affecting tea bud size, eight extreme accessions were selected from 280 accessions for comparative transcriptomics analyses, which included four small bud accessions (‘Baiye 1’ (BY1), ‘Taida’ (TD), ‘Qianfang’ (QF), ‘Guazijin’ (GZJ)) and four large bud accessions (‘Kuwei Baimudan’ (KWBMD), ‘Gelecha’ (GLC), ‘Chongpi 71–1’ (CP71–1), ‘Liercha’ (LEC); Fig. S1). Anatomical analysis of tea buds revealed significant differences from the middle layer to the outer layer (Fig. 2A). In the large bud group, the average bud length, width, perimeter, and area were 1.68 times, 1.70 times, 1.70 times, and 2.65 times larger than those in the small bud group, respectively (Fig. 2B). Overall, 153.58 G clean base were obtained by Illumina. The Average Q20, Q30, and GC content were 97.21%, 92.41%, and 42.82% respectively. Approximately 85.32% of the reads were mapped against the ‘Shuchazao’ V2 reference genome (Table S2) [35].

**Figure 3:**
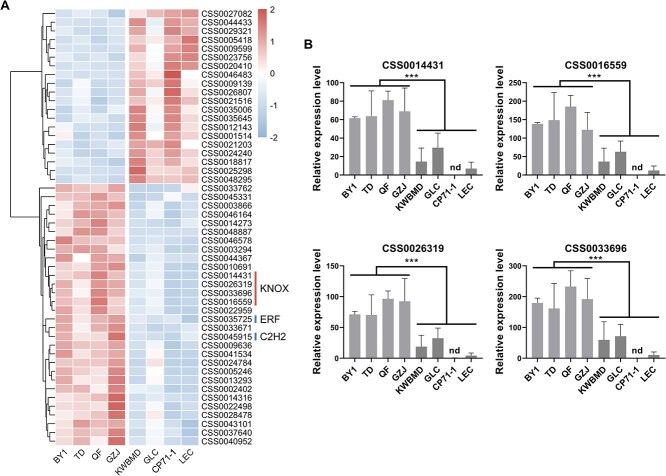
**The expression patterns of candidate genes and qRT-PCR validation.** (A) The clustering heat map displayed the expression patterns of 50 DEGs. Six TFs were labeled on the right side of the heat map. (B) The expression levels of *KNOX* genes were validated using qRT-PCR. Results are expressed as mean ± SEM (*n* = 3). Significant differences (*P* < 0.001) are indicated as ^***^. nd, not detected.

Pearson correlation analysis was performed on four traits related to bud size and gene expression levels. At *P* < 0.01 and |log_2_(Fold Change)| > 1, 97, 173, 107, and 127 genes were found for bud length, width, perimeter, and area, respectively (Fig. 2C). The Venn diagram indicated that there are 50 genes associated with the four traits (Fig. 2D, Table S3). Gene Ontology (GO) enrichment analysis was conducted on these genes, and the results showed that the top three terms with the highest gene counts are DNA binding, protein binding, and protein kinase activity (Fig. 2E). The clustering heat map displayed the expression patterns of 50 genes associated with bud size, including 20 upregulated genes and 30 downregulated genes (Fig. 3A). Six candidate TFs were screened out by expression pattern analysis, including four *KNOX* genes (CSS0014431, CSS0016559, CSS0026319, and CSS0033696)), one *C2H2* gene (CSS0045915), and one *bHLH* gene (CSS0045915). The expression levels of the four *KNOX* genes were validated using quantitative real-time polymerase chain reaction (qRT-PCR). The results indicated that the expression levels of the four *KNOX* genes in the large bud group were on average 5.34 times (CSS0014431), 5.31 times (CSS0016559), 5.91 times (CSS0026319), and 5.42 times (CSS0033696) higher than those in the small bud group (Fig. 3B). All of them showed a significant negative correlation with the phenotype, suggesting that these four genes may be candidate genes for negatively regulating tea bud size.

### Identification of candidate genes based on RNA-seq and GWAS

**Figure 4:**
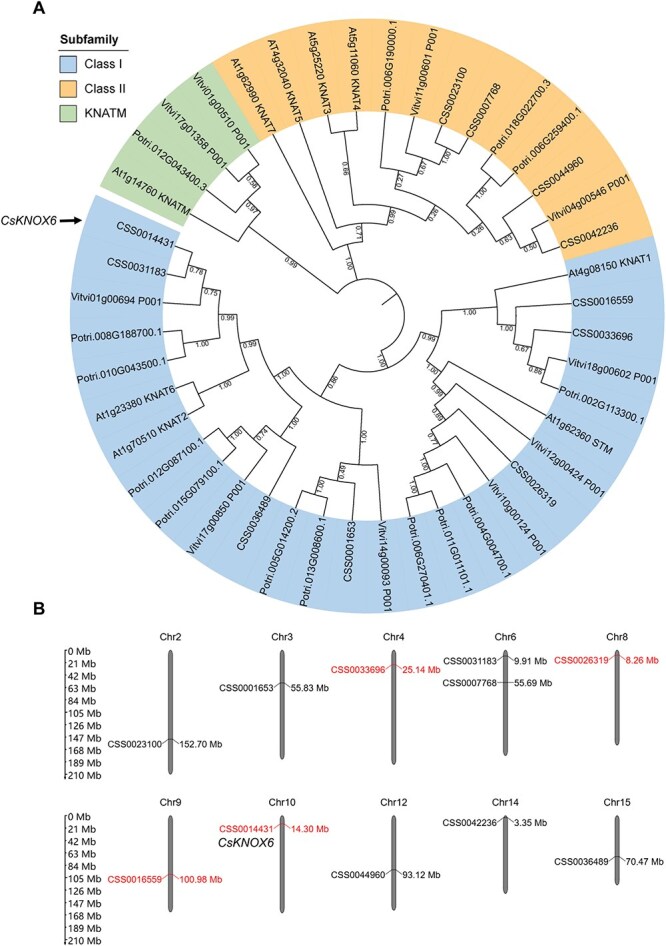
**Identification of *KNOX* genes in tea plant.** (A) Phylogenetic analysis of *KNOX* genes. The neighbor-joining phylogenetic tree was generated with the 1000 bootstrap values. (B) Chromosomal locations of the *KNOX* genes based on the genome of tea cultivar ‘Shuchazao’. Four candidate *KNOX* genes have been annotated on Chr4, Chr8, Chr9, and Chr10.

As mentioned before, due to the important role of *KNOX* genes in bud and leaf development [36], we screened for *KNOX* genes in the chromosomal-level genome of tea plant to further investigate their functions. Previous studies have suggested that the *KNOX* gene family primarily includes Class I, Class II, and KNATM subfamilies, with both Class I and Class II requiring four conserved domains (KNOX I, KNOX II, ELK, HD). The KNATM subfamily, on the other hand, contains only the KNOXI and KNOXII conserved domains and may have similar functions to Class I and Class II genes [29]. Based on the analysis of conserved domains, we identified a total of 11 *KNOX* genes in the tea plant genome and constructed a neighbor-joining phylogenetic tree using the amino acid sequences of *KNOX* genes from Arabidopsis, poplar, and grape (Fig. 4A). The results indicated that there are seven Class I genes and four Class II genes in the tea plant. Additionally, according to the definition of the KNATM subfamily, we found three genes in the tea plant genome. However, phylogenetic analysis showed that they did not cluster with the *KNATM* gene from Arabidopsis. This conclusion aligns with previous studies in terms of number but differs in specific classification counts [37]. Notably, all four candidate *KNOX* genes belong to the Class I subfamily. According to the genomic location information, these 11 *KNOX* genes are distributed across 10 chromosomes, with two *KNOX* genes located on Chr6, while one *KNOX* gene each is found on Chr2, Chr3, Chr4, Chr8, Chr9, Chr10, Chr12, Chr14, and Chr15 (Fig. 4B). The four candidate *KNOX* genes are located on Chr4 (CSS0033696), Chr8 (CSS0026319), Chr9 (CSS0016559), and Chr10 (CSS0014431).

**Figure 5:**
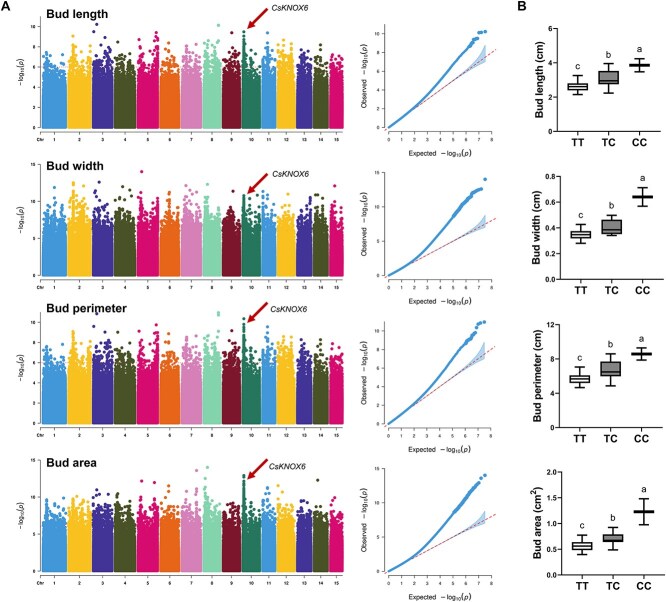
**GWAS analysis of four tea bud traits.** (A) Manhattan plot and Quantile–quantile (QQ) plot of the GWAS analysis of bud length, width, perimeter, and area. The dotted lines are the threshold level (−log_10_P = 6). The positions indicated by the arrows represent the candidate interval containing *KNOX* gene CSS0014431. (B) Box plot of four bud trait values among the 280 tea accessions at the lead GWAS SNP (Chr10:14322573). Different letters indicate significant difference (*P* < 0.05) according to Tukey’s HSD test.

To further clarify the key *KNOX* genes, GWAS analysis was conducted on four traits of 280 tea accessions using TeaGVD [38] (Fig. 5A). A total of 688 candidate intervals were identified, covering a range of 155.99 Mb, which included 2909 candidate genes (Table S4). Among the four *KNOX* genes, only *CsKNOX6* (CSS0014431) was located within the candidate interval from 14.22 to 14.42 Mb on Chr10. When stratified by genotype, the phenotypic values of bud length, width, perimeter, and area among tea accessions mentioned above could be clearly distinguished at lead single nucleotide polymorphism (SNP) (Chr10:14322573) (Fig. 5B). Therefore, *CsKNOX6* (CSS0014431) was identified as a key gene influencing tea bud size. In addition to the *KNOX* family genes, several other candidate genes associated with bud size were identified through homologous gene function and expression pattern analysis, providing further insights into the genetic regulation of bud size. Within the candidate region on Chr12 (Chr12: 140.68–140.88 Mb), CSS0017314 was identified, which is homologous to AT2G45190 in Arabidopsis belonging to the YAB family of TFs. *YAB* genes, along with *KNOX* family genes, are known to play crucial roles in regulating leaf morphogenesis and lateral organ development in Arabidopsis [39]. CSS0015300 was identified within the region on Chromosome 4 (Chr4: 177.48–177.68 Mb). This gene is homologous to *AS1* in Arabidopsis, a member of the MYB TF family. AS1 has been well characterized for its role in controlling leaf cell differentiation and maintaining leaf polarity [40]. Its association with bud size in tea plants implies that it may play a role in regulating cell proliferation and differentiation during bud development.

### Functional validation of *CsKNOX6*

**Figure 6:**
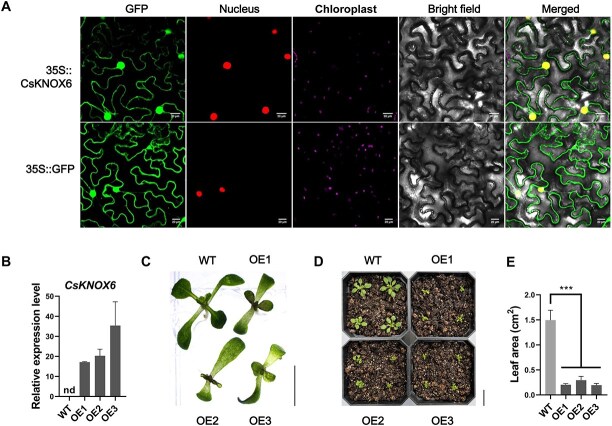
**Functional validation of *CsKNOX6*.** (A) Subcellular localization of CsKNOX6 protein in transformed *Nicotiana benthamiana* leaves. The first, second, and third columns indicate the locations of the GFP fusion protein, the nucleus, and the chloroplasts, respectively. The 35S::GFP empty vector was used as controls. White bar = 20 μm. (B) Expression levels of *CsKNOX6* in T_3_-generation homozygous overexpressing Arabidopsis (OE1, OE2, and OE3) and WT plants. Results are expressed as mean ± SEM (*n* = 3). nd, not detected. (C) Phenotypes of 7-day-old transgenic Arabidopsis and WT plants. Black bar = 0.5 cm. (D) Phenotypes of 14-day-old transgenic Arabidopsis and WT plants. Black bar = 2 cm. (E) Leaf area of transgenic and WT Arabidopsis after 14 days of growth. Results are expressed as mean ± SEM (*n* = 4). Significant differences (*P* < 0.001) are indicated as ^***^.

Based on the reference genome sequence, full-length primers were designed to amplify the CDS of CSS0014431, resulting in a sequence of 951 bp that encodes a protein of 316 amino acids. Since this gene has the highest homology with *KNAT6* in Arabidopsis, it was tentatively designated as *CsKNOX6*. Subcellular localization results indicate that this protein is located in the nucleus, which is consistent with the typical localization of TFs (Fig. 6A) [41]. Due to the current immaturity of the transgenic system in tea plants, we therefore overexpressed *CsKNOX6* in Arabidopsis to explore its potential function. Through the floral dip method, we successfully obtained three homozygous T_3_-generation *CsKNOX6*-OE lines (OE1, OE2, and OE3), which exhibited higher expression levels of *CsKNOX6* compared to the wild type (WT) (Fig. 6B). Compared to the WT, the leaf rosettes of 7-day-old overexpressing lines exhibited developmental abnormalities, and the leaves were significantly smaller than those of the WT (Fig. 6C). The leaf area of 14-day-old overexpressing lines were also significantly (*P* < 0.001) smaller than that of the WT (Fig. 6D). The leaf areas of OE1, OE2, and OE3 were only 13.61%, 19.71%, and 13.09% of that of the WT, respectively. (Fig. 6E). Overall, our data showed that overexpression of *CsKNOX6* in Arabidopsis resulted in a reduction in leaf area, which further indicates that this gene may be closely associated with the size of tea buds.

## Discussion

Tea plants are important leaf-based economic crops, with new shoots as the primary target for harvesting [42]. The traits associated with new shoots, such as the size, color, and trichomes of the buds and leaves, directly influence the yield and quality of tea, thereby affecting economic benefits [43]. Bud size is a significant agronomic and biological trait of tea plants; it not only impacts yield but is also closely related to the suitability of different tea types [44]. Different types of tea have distinct appearances, leading to varying requirements for bud and leaf size.

The buds of the tea plant are categorized into two types: leaf buds and flower buds. The tea buds harvested in production are leaf buds, also known as vegetative buds, which can develop into branches and leaves [45]. The shoots formed from these buds are the main target for harvest, and their tissue structure includes the SAM, leaf primordia, and young leaves [46]. Therefore, tea buds undergo a growth and development process similar to that of plant leaves, suggesting that bud size is influenced by genetic regulatory mechanisms similar to those affecting leaf size. Although ecological environment and cultivation techniques influence plant leaf traits to some extent, variety remains the decisive factor [47]. With the advancement of image processing technology, it is now possible to achieve higher throughput and greater accuracy in plant phenotyping [48]. In this study, we used image processing technology to identify the bud size phenotypes of 280 accessions of tea germplasm. We clarified the distribution, variation patterns, and genetic diversity of bud size traits within natural populations. All four phenotypic traits exhibited a normal distribution in the natural population, indicating that bud size is a typical quantitative trait regulated by multiple genes [49, 50]. The four phenotypic traits demonstrated high heritability (h^2^ > 0.6), further confirming that bud size is minimally affected by environmental factors [51], with different tea plant cultivars being the primary influence on bud size. The results presented in this study are based on phenotypic data collected from a single year, which limits our ability to account for annual environmental variations that significantly influence bud size. The lack of multiyear data may affect the accuracy of phenotypic characterization and the robustness of the identified genetic associations. Future studies incorporating annual repetitions across diverse environments are necessary to validate these findings and assess the stability of the key loci underlying bud size.

GWAS is an analytical method based on the principle of linkage disequilibrium, used to detect associations between genetic variations and phenotypic traits [52]. Unlike linkage mapping methods, its subjects typically involve natural populations with unstable structure and rich genetic diversity. As a result, leveraging the vast genetic variation found in natural populations allows for more precise mapping and may even lead to the direct identification of the genes themselves [53, 54]. With the release of the chromosome-level reference genome of tea plant, GWAS analysis based on the timing of spring bud flush have been widely conducted [55–57]. In the study of tea leaf phenotypes, Niu *et al.* conducted GWAS analysis on 415 tea accessions from four provinces across China using 30 282 high-quality SNPs. They identified nine SNPs significantly associated with leaf size and demonstrated that leaf size is regulated by major effect genes [58]. Similar conclusions were also found in SSR-based genetic mapping and QTL analysis of mature tea leaf size [59]. Chen *et al.* conducted GWAS analysis on 338 tea accessions from Guizhou using 100 829 high-quality SNPs obtained from GBS sequencing, identifying six candidate genes related to leaf size, texture, color, and shape. Among these, two candidate genes on chromosomes Chr1 and Chr9 were significantly associated with leaf size, belonging to the tetratricopeptide-like helix domain superfamily and phosphofructokinase superfamily, respectively [60].

In this study, we conducted transcriptome analysis of eight extreme materials and identified 50 genes associated with bud size, including four differentially expressed *KNOX* genes, all belonging to Class I *KNOX* genes. Existing research indicates that this class is closely related to the morphological development of plant buds and leaves [61–63]. The expression trends of these four KNOX genes are consistent and show a significant negative correlation with bud size, which aligns with findings that overexpression of *KNOX* genes in citrus and agave leads to smaller leaves [64, 65]. Further GWAS analysis results suggest that *CsKNOX6* may be a key gene responsible for the differences in bud size among different tea cultivars. This gene is located at the top of Chromosome 10 and encodes 316 amino acids. Subcellular localization results show that this gene is situated in the nucleus, consistent with the localization of most TFs [66, 67]. Overexpression validation in Arabidopsis revealed that overexpression of this gene leads to abnormal leaf development in seedlings, resulting in significantly smaller leaves, further indicating that *CsKNOX6* may be a critical negative regulator of tea bud size. While the Arabidopsis system provides valuable preliminary insights, the observed phenotypic effects in Arabidopsis may not fully recapitulate the role of *CsKNOX6* in tea plants due to species-specific differences in gene regulatory networks and developmental programs. For example, the function of *KNOX* genes in apical bud development may vary significantly between herbaceous plants like Arabidopsis and perennial woody plants like tea. In the future, these findings should be further validated in tea plants or other horticultural plants with well-defined apical buds using advanced genetic tools, such as CRISPR/Cas9-mediated gene editing or stable transformation.

## Conclusions

This study utilized image recognition technology to collect images of the apical buds at the one bud and two leaves stage from 280 representative tea accessions. Genetic diversity analysis indicated that the length, width, perimeter, and area of tea buds all displayed a normal distribution. Comparative transcriptomic analysis of extreme materials revealed a significant negative correlation between the expression levels of four *KNOX* genes and tea bud size. Further GWAS analysis and Arabidopsis overexpression validation suggested that *CsKNOX6* may be a key gene negatively regulating tea bud size. These conclusions have potential significance for tea plant breeding programs and increasing tea yield.

## Materials and methods

### Plant materials

The experimental materials were sourced from the National Tea Germplasm Repository at Hangzhou in the TRICAAS, China. The 280 tea accessions of representative germplasm included wild relatives, landraces, and modern cultivars. The sampling period was from 29 March to 15 April 2023. Each accession was collected at the one bud with two leaves stage, with at least 15 apical buds sampled from each accession. Information on the 280 tea accessions was provided in Table S1. Fresh apical buds from eight extreme accessions representing bud size phenotypes were collected, including four small bud accessions (‘BY1’, ‘TD’, ‘QF’, and ‘GZJ’) and four large bud accessions (‘KWBMD’, ‘GLC’, ‘CP71–1’, and ‘LEC’). These samples were then stored at −80°C for RNA extraction.

### Image collection and feature extraction

The Canon LiDE 400 portable scanner was used as the imaging tool. The harvested tea plant apical buds were evenly laid on the scanner’s flatbed, with black light-absorbing cloth chosen as the scanning background. Images were scanned using the Canon IJ Scan Utility Lite (Ver. 3.3.1), with the following scan option parameters set: source ‘Photo’, color mode ‘Color’, paper size ‘Full Document’, resolution ‘600 dpi’, and data format ‘PNG’. Each sample was scanned to obtain one image, with each image measuring 5100 × 7016 pixels.

Using the leafAI software (https://github.com/JDChenTea/leafAI), the collected apical bud images were processed for feature extraction. The software is based on the Python libraries OpenCV and NumPy. First, the tea plant apical bud images were segmented to obtain individual RGB images of the buds and extract their color features. Further image preprocessing was conducted to obtain grayscale and binary images, extracting texture and morphological features. Finally, four image features related to bud size phenotypes were selected: bud length, width, perimeter, and area. The data was extracted and saved for subsequent image feature analysis. A comprehensive validation by comparing our digital measurements with manual measurements was performed. The result demonstrated an exceptionally high consistency of bud length between the two methods, with an R^2^ value of 0.99 and an RMSE of 0.07 (Fig. S2).

Statistical analysis of the extracted phenotypic data was performed using Excel software. The maximum and minimum values of four image features were calculated, along with the mean, standard deviation (SD), CV, diversity index (*H′*), and broad-sense heritability (*h*^2^). Hierarchical clustering of 280 accessions was performed using the hclust function in R. A dendrogram was plotted using the R package factoextra (Ver. 1.0.7).

### RNA-seq

Total RNAs were extracted with the EASY-spin Plus Complex Plant RNA Kit (Ailab, Beijing, China) following the manufacturer’s recommendations. Sequencing libraries were generated on Illumina Novaseq 6000 platform with PE150 strategy using a customer sequencing service (Novogene, Beijing, China). Clean reads were obtained by removing reads containing adapter contamination and low-quality bases from raw data with Sickle [68]. The index of the reference genome was built and paired-end clean reads were aligned to the reference genome with the HISAT2 [69]. Assemble the reads that aligned to the reference genome using StringTie [70] and map them to potential transcripts. The prepDE.py script was used for quantifying transcript expression and output a gene expression matrix. Transcripts per kilobase of exon model per million mapped reads (TPM) were used to quantify transcript abundance. The Pearson correlation coefficient between bud size and gene expression was evaluated by cor.test function in R. Genes associated with bud size were identified based on a *P-*value of the Pearson correlation coefficient <0.01 and |log_2_(Fold Change)| > 1. The clusterProfiler R package (Ver. 4.6.2) was applied to GO enrichment analysis [71]. The heat map was generated with pheatmap R package (Ver. 1.0.12) [72].

### Quantitative real-time PCR analysis

The expression levels of candidate genes were validated by qRT-PCR. The cDNA was reversely transcribed using PrimeScript™ RT reagent Kit (Takara, Dalian, China). qRT-PCR primers of candidate genes were designed based on the tea plant genome information. The specific primer information is provided in Table S3. Relative gene expression was calculated by the 2^-ΔΔCt^ method, with *CsGAPDH* (accession no. KA295375) as the reference gene.

### Genome-wide association analysis

A genome-wide association study of bud size traits, including bud length, bud width, bud perimeter, and bud area, was performed by a mixed linear model implemented EMMAX software based on SNP information of 280 accessions from TeaGVD database [38, 73]. A threshold of *P* < 6 × 10^−6^ was used to identify genome-wide significant loci. A locus was considered as a candidate SNP-related bud size if it was significantly associated with at least two of the traits: bud length, bud width, bud perimeter, and bud area. A 100-kb interval flanking the candidate SNP was designated as the candidate interval, and overlapping regions were merged using Bedtools.

### Gene cloning of *CsKNOX6*

Full-length primers for *CsKNOX6* were designed based on the sequence information of the ‘Suchazao’ reference genome (Table S3). The cDNA from the tea cultivar ‘GZJ’ was used as the template for gene cloning. PCR amplification was performed using the KOD-Plus-Neo enzyme (Toyobo, Osaka, Japan), and the conditions were as follows: 2 min at 94°C, 33 cycles of 94°C for 10 s, 58°C for 30 s, and 68°C for 30 s, and a final extension at 68°C for 5 min. The PCR product was cloned into the *pEASY*-Blunt Zero Cloning vector (TransGen Biotech, Beijing, China), transformed into *Escherichia coli Trans*1-T1 competent cells, and confirmed by sequencing.

### Subcellular localization of CsKNOX6

For protein subcellular localization observation, *CsKNOX6* gene was cloned and inserted into the pBWA(V)BS vector. The recombinant vector was then transformed into *Agrobacterium tumefaciens* GV3101. Transient expression of fluorescent fusion proteins in tobacco plants was carried out according to a published method [74]. The p35S::GFP empty vector were used as controls. The GFP fluorescent signal was observed 2 days after the infiltration using a Nikon C2-ER confocal laser scanning microscope (Nikon, Tokyo, Japan) with excitation wavelength of 488 nm. The mKTAE with nuclear localization sequences (MDPKKKRKV) was used as a nuclear marker, with excitation wavelength of 561 nm [75], and the excitation wavelength for chloroplast autofluorescence was 640 nm.

### Function verification of *CsKNOX6* in Arabidopsis

For *CsKNOX6* function verification, *A. tumefaciens* GV3101 containing the pBWA(V)BS overexpression vector was used to infect the Arabidopsis Col-0 according to the standard flower dipping method [76]. The T_3_-generation homozygous lines were verified by qRT-PCR and used for further analysis. Relative gene expression of *CsKNOX6* in overexpressing Arabidopsis was calculated by the 2^-ΔCt^ method using the *AtTUB2* sequence (AT5G62690) as the reference gene. The transgenic and WT Arabidopsis were germinated on half-strength MS medium for 7 days under a 16 h light/8 h dark photoperiod at 25°C and the phenotypes were observed. The Arabidopsis plants were then transplanted into nutrient soil and continued to be cultured for 7 days. Leaf area was calculated using ImageJ software [77].

## Supplementary Material

Web_Material_uhaf051

## Data Availability

The clean sequencing data for this study can be found in the CNCB (www.cncb.ac.cn) Genome Sequence Archive (GSA) under the BioProject accession no. PRJCA031997.
