# Supplementary material for: Integration of digital phenotyping, GWAS, and transcriptomic analysis revealed a key gene for bud size in tea plant (Camellia sinensis)
Source: Hortic Res. 2025 Feb 20;12(6):uhaf051. doi: 10.1093/hr/uhaf051 (PMC12015473; doi:10.1093/hr/uhaf051)
Supplement: Web_Material_uhaf051 [file web_material_uhaf051.zip › Supplementary Figure.docx]

**Supplementary Figure**


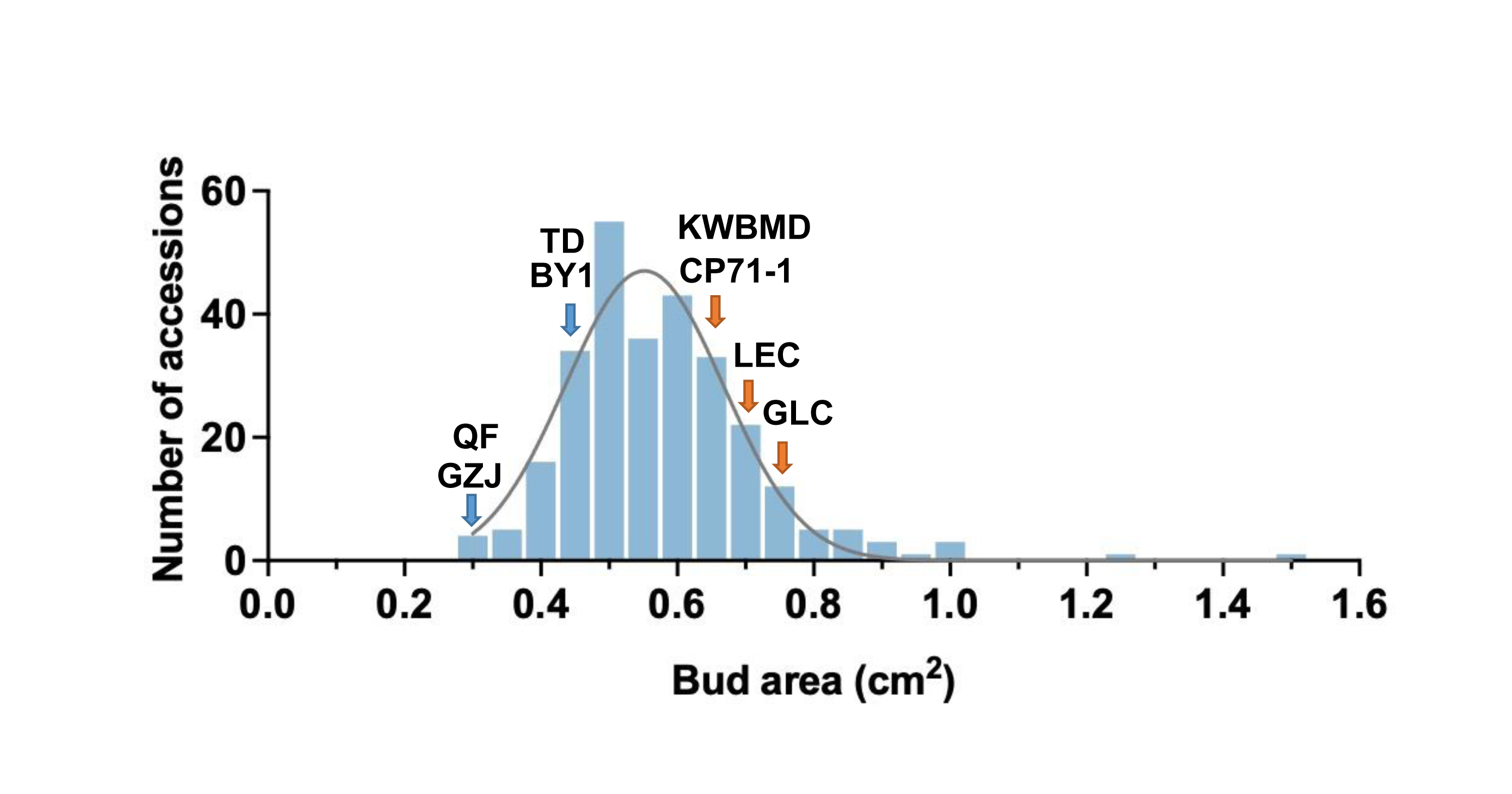


**Supplementary** **Figure S1.** Frequency distribution of bud area and extreme accessions.


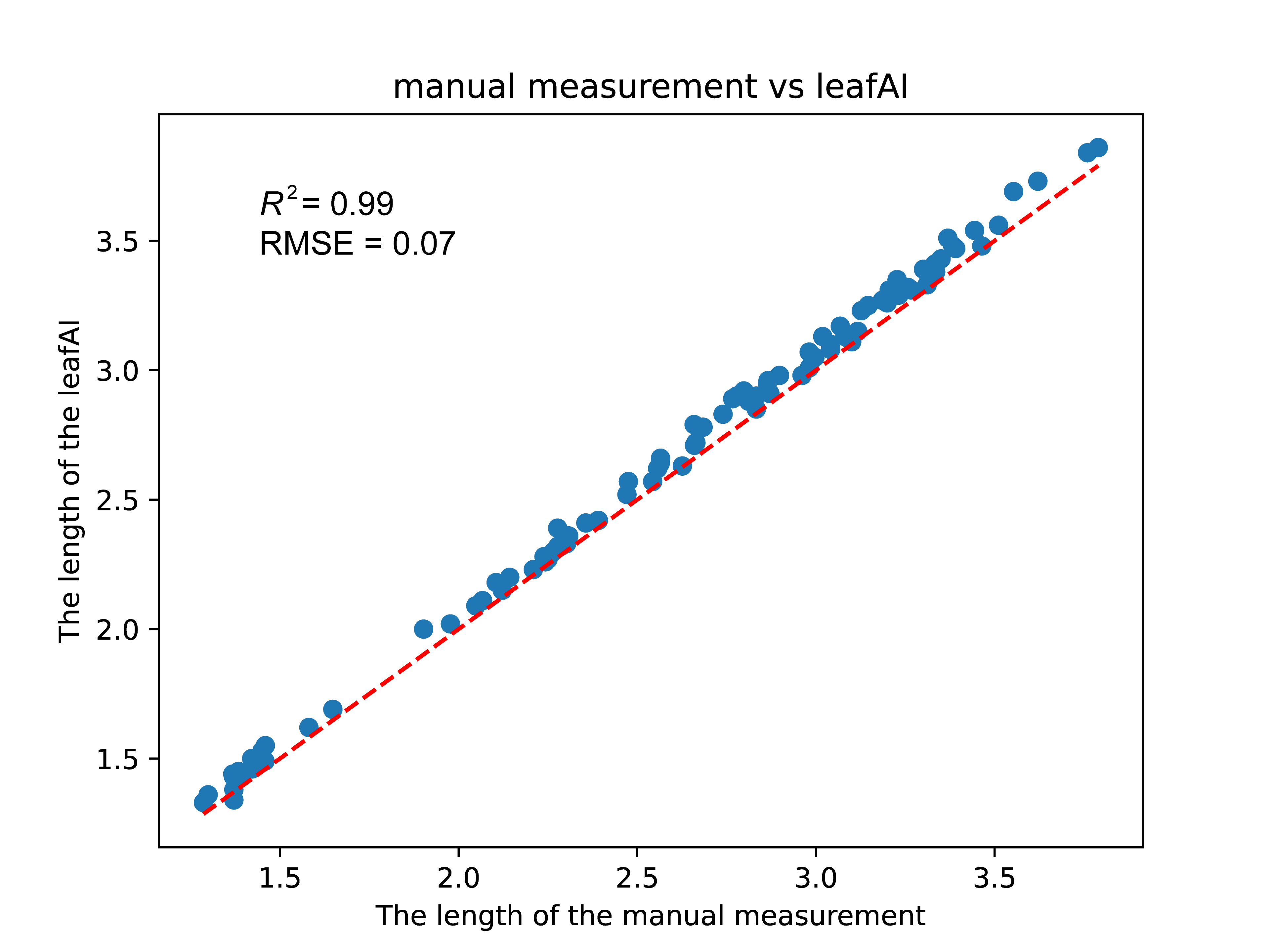


**Supplementary Figure S2.** Validation of the digital measurement for bud length on the basis of the manual measurement dataset.
